# Skin-associated lactic acid bacteria from North American bullfrogs as potential control agents of *Batrachochytrium dendrobatidis*

**DOI:** 10.1371/journal.pone.0223020

**Published:** 2019-09-27

**Authors:** M. V. Niederle, J. Bosch, C. E. Ale, M. E. Nader-Macías, C. Aristimuño Ficoseco, L. F. Toledo, A. Valenzuela-Sánchez, C. Soto-Azat, S. E. Pasteris

**Affiliations:** 1 Instituto Superior de Investigaciones Biológicas (INSIBIO), Consejo Nacional de Investigaciones Científicas y Técnicas (CONICET) and Instituto de Biología “Dr. Francisco D. Barbieri”, Facultad de Bioquímica, Química y Farmacia, Universidad Nacional de Tucumán (UNT), San Miguel de Tucumán, Argentina; 2 Museo Nacional de Ciencias Naturales, CSIC, Madrid, Spain; 3 Research Unit of Biodiversity (CSIC, UO, PA), Oviedo University—Campus Mieres, Spain; 4 Centro de Referencia para Lactobacilos (CERELA), Consejo Nacional de Investigaciones Científicas y Técnicas (CONICET), San Miguel de Tucumán, Argentina; 5 Laboratório de História Natural de Anfíbios Brasileiros (LaHNAB), Departamento de Biologia Animal, Instituto de Biologia, Universidade Estadual de Campinas, Campinas, São Paulo, Brazil; 6 Centro de Investigación para la Sustentabilidad, Facultad de Ciencias de la Vida, Universidad Andres Bello, Santiago, Chile; 7 Instituto de Ciencias Ambientales y Evolutivas, Facultad de Ciencias, Universidad Austral de Chile, Valdivia, Chile; 8 Organización No Gubernamental (ONG) Ranita de Darwin, Santiago, Chile; 9 Organización No Gubernamental (ONG) Ranita de Darwin, Valdivia, Chile; Universite Paris-Sud, FRANCE

## Abstract

The fungal pathogen *Batrachochytrium dendrobatidis* (*Bd*) is the causative agent of chytridiomycosis and has been a key driver in the catastrophic decline of amphibians globally. While many strategies have been proposed to mitigate *Bd* outbreaks, few have been successful. In recent years, the use of probiotic formulations that protect an amphibian host by killing or inhibiting *Bd* have shown promise as an effective chytridiomycosis control strategy. The North American bullfrog (*Lithobates catesbeianus*) is a common carrier of *Bd* and harbours a diverse skin microbiota that includes lactic acid bacteria (LAB), a microbial group containing species classified as safe and conferring host benefits. We investigated beneficial/probiotic properties: anti-*Bd* activity, and adhesion and colonisation characteristics (hydrophobicity, biofilm formation and exopolysaccharide-EPS production) in two confirmed LAB (cLAB-*Enterococcus gallinarum* CRL 1826, *Lactococcus garvieae* CRL 1828) and 60 presumptive LAB (pLAB) [together named as LABs] isolated from bullfrog skin.We challenged LABs against eight genetically diverse *Bd* isolates and found that 32% of the LABs inhibited at least one *Bd* isolate with varying rates of inhibition. Thus, we established a score of sensitivity from highest (*Bd*GPL AVS7) to lowest (*Bd*GPL C2A) for the studied *Bd* isolates. We further reveal key factors underlying host adhesion and colonisation of LABs. Specifically, 90.3% of LABs exhibited hydrophilic properties that may promote adhesion to the cutaneous mucus, with the remaining isolates (9.7%) being hydrophobic in nature with a surface polarity compatible with colonisation of acidic, basic or both substrate types. We also found that 59.7% of LABs showed EPS synthesis and 66.1% produced biofilm at different levels: 21% weak, 29% moderate, and 16.1% strong. Together all these properties enhance colonisation of the host surface (mucus or epithelial cells) and may confer protective benefits against *Bd* through competitive exclusion. Correspondence analysis indicated that biofilm synthesis was LABs specific with high aggregating bacteria correlating with strong biofilm producers, and EPS producers being correlated to negative biofilm producing LABs. We performed Random Amplified Polymorphic DNA (RAPD)-PCR analysis and demonstrated a higher degree of genetic diversity among rod-shaped pLAB than cocci. Based on the LAB genetic analysis and specific probiotic selection criteria that involve beneficial properties, we sequenced 16 pLAB which were identified as *Pediococcus pentosaceus*, *Enterococcus thailandicus*, *Lactobacillus pentosus*/*L*. *plantarum*, *L*. *brevis*, and *L*. *curvatus*. Compatibility assays performed with cLAB and the 16 species described above indicate that all tested LAB can be included in a mixed probiotic formula. Based on our analyses, we suggest that *E*. *gallinarum* CRL 1826, *L*. *garvieae* CRL 1828, and *P*. *pentosaceus* 15 and 18B represent optimal probiotic candidates for *Bd* control and mitigation.

## Introduction

Amphibians play an important ecological role in the transport of energy from aquatic environments to terrestrial ecosystems, and several factors have been proposed to contribute to their population declines worldwide [1,2]. Among the threats to amphibian survival, the most commonly cited are habitat loss, pollution, the pet trade, climate change, and emerging infectious diseases (EID) [3]. Chytridiomycosis is a major amphibian EID caused by two congeneric species of chytrid fungi: *Batrachochytrium salamandrivorans* [4] and *B*. *dendrobatidis* (*Bd*) [5–8]. In anurans, *Bd* proliferates in the keratinized epithelial cells of post-metamorphic animals and the mouthparts of tadpoles [9] with mortality occurring due to osmotic imbalance and subsequent asystolic cardiac arrest [10]. In addition, *Bd* has been shown to inhibit normal lymphocyte function and proliferation and disturb cellular energy pathways [11,12].

To date, *Bd* control in nature has proven difficult [13] since amphibian populations can host multiple *Bd* genotypes, such as *Bd*GPL (Global Panzootic Lineage), *Bd*Hybrid lineages, as well as endemic lineages [14,15]. There is only one effective practical intervention eradicating *Bd* based on antifungals [16], thus probiotics represent an attractive alternative tool for *Bd* control in nature [17–19] with potential advantages over antifungal drugs, which are difficult to apply in the wild and may have profound effects to the native microbiota of a host or ecosystem [16,20]. In aquaculture, probiotics may confer benefits to the health of their host or the environment through different modes of action including antagonistic activity against pathogens [15,20–28], enhanced competitive exclusion of pathogens through increased host microbial load and diversity [15,17,29], modulation of pathogen virulence, adhesion to host epithelial cells, stimulation of the immune response [17,30–32] and improvement of water quality [33].

In amphibians, the first line of defence against pathogens is the skin, where two key protective mechanisms may operate: the microbiota associated with the cutaneous structures (epithelial cells and mucus) [34], and the antimicrobial peptides produced by glandular glands and secreted within the host’s skin [35]. Bacterial species of the amphibian skin microbiome can provide protection from *Bd* infection through competition for nutrients and chemotactic factors [36,37], as well as through the production of antifungal metabolites [21]. Several *in vitro* studies have reported anti-*Bd* activity of skin-associated Gram-negative and some Gram-positive bacteria isolated from different amphibian species (**Table 1**). Based on the ability of bacteria to produce antifungal metabolites (such as violacein, prodigiosin, 2,4-diacetylphloroglucinol, indole-3-carboxaldehyde) some bacterial strains [20–28] or species combinations (e.g. production of tryptophol in *Bacillus* sp. and *Chitinophaga arvensicola* mixed communities) [38] have been selected as potential probiotics to mitigate *Bd* infection and chytridiomycosis development.

**Table 1:** Bacterial species and some of its identified anti-*Batrachochytrium dendrobatidis* (*Bd*) metabolites.

Phylogenetic lineage	Species	Anti-*Bd* metabolite	Reference
Gammaproteobacteria	*Lysobacter gummosus*	2,4-diacetylphloroglucinol	[20,22]
	*Stenotrophomonas maltophilia*	None assigned	[21,27]
	*Serratia plymuthica*	Prodigiosin	[21]
	*Serratia marcescens*	Prodigiosin	[21]
	*Pseudomonas* sp.	None assigned	[27]
	*Pseudomonas mosselii*	None assigned	[26]
	*Pseudomonas fluorescens*	None assigned	[38]
Betaproteobacteria	*Janthinobacterium lividum*	Violacein, indole-3-carboxaldehyde	[21]
	*Delftia tsuruhatensis*	None assigned	[26]
Actinobacteria	*Arthrobacter* sp.	None assigned	[26]
	*Streptomyces* sp.	None assigned	[26]
	*Kitasatospora* sp.	None assigned	[26]
Firmicutes	*Bacillus* sp.	None assigned	[26,27]
	*Paenibacillus* sp.	None assigned	[26]
Bacteroidetes / Chlorobium	*Chryseobacterium jejuense*	None assigned	[26]
	*Chryseobacterium antarcticum*	None assigned	[26]
	*Chryseobacterium indologenes*	None assigned	[26]

The normal microbiota of North American bullfrog (*Lithobates catesbeianus*; from here on referred as “bullfrog”) skin in hatchery conditions is known to include Enterobacteriaceae (*Citrobacter freundii*, *Enterobacter* spp., *Escherichia coli*, *E*. *blattae*, *Klebsiella* spp., *Proteus vulgaris*), *Pseudomonas aeruginosa*, *Staphylococcus epidermidis*, *Bacillus* spp., *Micrococcus* spp. and Lactic Acid Bacteria (LAB) [39–43]. This last group is specifically classified as Gram-positive, catalase and oxidase negative, indol and nitrate negative, non-sporulating and usually non-motile microorganisms. According to studies performed in different niches, LAB comprise the following genera: *Carnobacterium*, *Dolosigranulum*, *Lactobacillus* (rods); *Aerococcus*, *Alloiococcus*, *Enterococcus*, *Lactococcus*, *Leuconostoc*, *Oenococcus*, *Pediococcus*, *Streptococcus*, *Tetragenococcus*, *Vagococcus* (cocci), and *Weissella* (coccoid or rod-shaped) [44–47].

On the basis of their Generally Regarded as Safe (GRAS) properties (no translocation ability, absence of virulence factors, no antibiotic resistance) [48], Food Grade characteristics and Qualified Presumption as Safety (QPS) for the European Food Safety Authority (EFSA) [49], some LAB species have been proposed as probiotics in aquaculture for infectious diseases control and improvement of zootechnic parameters (animal growth and nutrition) [50–52]. These microorganisms produce a range of antimicrobial metabolites such as organic acids, bacteriocins, diacetyl, and hydrogen peroxide. Bacteriocins are known to inhibit other LAB strains, some Gram-negative pathogens and Gram-positive spoilage bacteria [53,54], as well as moulds and yeasts [55,56].

A broad range of microbial characteristics such antimicrobial activity and adhesion/colonisation properties must be considered when selecting microorganisms to be included in a probiotic formula [48]. In particular, key properties include the hydrophobicity of the bacterial cell surface that can impact bacterial adhesion to skin epithelial cells [57], while auto-aggregation (an interaction phenomenon that occur between microorganisms of the same strain) [58], exopolysaccharide production and biofilm formation [52,59,60] can impede pathogen colonisation of the host skin [61].

Ranaculture is a branch of aquaculture that involves raising amphibians for commercial purposes. The bullfrog is the most globally reared amphibian species and is grown to provide meat while by-products such as the skin are used as a source of compounds in human antitumor therapy [62]. However, bullfrogs are vulnerable to bacterial infection diseases in hatcheries conditions and are also well known for its *Bd* carrier capability [63–66]. Since LAB are commonly found in the native microbiota of bullfrog hatcheries and some strains have previously been selected as probiotic candidates for control of Red-Leg Syndrome (RLS) [39–43], we evaluated their potential as probiotics by measuring the inhibitory activity of confirmed LAB (cLAB) and presumptive LAB (pLAB) from bullfrog skin on *Bd* isolates from multiple lineages, in addition to properties related to host adhesion and colonisation. Taking into account that microorganisms intended for inclusion in a probiotic product must be correctly identified [67], we carried out genotypic characterization of selected isolates as well as compatibility assays for the potential formulation of mixed probiotic consortia.

This study contributes to our understanding of probiotic design and demonstrates a potential future use of GRAS microorganisms for *Bd* control *in situ* and during the *ex situ* breeding of endangered amphibian species.

## Material and methods

### Microorganisms and culture conditions

For the all assays, unless otherwise stated, we used *Enterococcus gallinarum* CRL 1826, *Lactococcus garvieae* CRL 1828 (confirmed LAB-cLAB) and 60 presumptive LAB (pLAB) [together named as LABs, n = 62]. All bacteria were previously isolated from ventral and dorsal skin areas of captive bullfrogs in the fattening phase of growth in a hatchery located in central Argentina (Río Cuarto, Córdoba) [43]. All pLAB were classified based on staining (Gram-positive) and key biochemical properties (catalase negative, nitrate and indol negative) [43]. All LABs were grown in de Man, Rogosa and Sharpe broth (MRS in g/L: peptone, 10; meat extract, 10; yeast extract, 5; glucose, 20; sodium acetate, 5; triammonium citrate, 2; K_2_HPO_3_, 2; MgSO_4_.7H_2_O, 0,2; MnSO_4_.4H_2_O, 0,05; polyoxyethylene sorbitan mono-oleate-Tween 80, 1 mL) [68], pH 6.8 at 37°C for 12 h and then adapted by subsequent culture (72 h) in TG (16 g/L tryptone + 1 g/L glucose) broth, pH 7.0. In both culture media, the microorganisms were incubated in microaerophilia (5% CO_2_ atmosphere). For anti-*Bd* assays, we used eight *Bd* isolates belonging to hypervirulent, hypovirulent and hybrid lineages (**Table 2**). The isolates were cryopreserved in liquid nitrogen and recovered after two passages on tryptone-glucose-agar (0.9% w/v) (TGA) at 20°C for 7 to 10 days.

**Table 2:** *Batrachochytrium dendrobatidis* (*Bd*) isolates and their geographical origin.

*Bd* isolate	Genetic lineage	Geographical origin	Reference
UM142	*Bd*ASIA-2/*Bd*Brazil	Ypsilanti, Michigan, USA	[66]
CLFT001	*Bd*ASIA-2/*Bd*Brazil	Jundiaí, São Paulo, Brazil	[69]
CLFT024.02	*Bd*Hybrid	Estrada da Graciosa, Morretes, Paraná, Brazil	[66]
CLFT159	*Bd*GPL	Estrada da Graciosa, Morretes, Paraná, Brazil	[70]
AVS4	*Bd*GPL	Hualañé, Región Maule, Chile	[71]
AVS7	*Bd*GPL	Valdivia, Región Los Ríos, Chile	[71]
C2A	*Bd*GPL	Peñalara Massif, Sierra de Guadarrama National Park, Madrid, Spain	[72]
VA02	*Bd*GPL	Valencia, Spain	[72]

### Anti-*Batrachochytrium dendrobatidis* (*Bd*) activity of confirmed and presumptive lactic acid bacteria (LABs)

We evaluated the anti-*Bd* activity of LABs using co-culture assays. Thus, after 4 days of *Bd* growth (maximum zoospore production), *Bd* plates were flooded with 3 mL of tryptone broth. After 20 min, the plates were flooded again and left to sit for another 20 min. The resulting liquid was then filtered through sterilized nylon (20 μm) and zoospore density was determined using a haemocytometer [23]. We then inoculated 500 μL of 2.7x10^6^ *Bd* zoospores/mL on TGA and spread with a Drigalsky's spatula to produce a “lawn”. The plates were allowed to dry in a laminar flow hood until they were slightly moist and 10 μL of 1x10^5^ CFU/mL LABs were streaked across the plates in a straight line and incubated for 7 to 10 days at 20°C. The score for antimicrobial activity was adapted from Park et al. [26] as follows: (A) no antifungal activity: the whole plate was evenly covered with *Bd* growth; (B) low antifungal activity: a minimal zone of zoospore inhibition; (C) medium antifungal activity: an asymmetrical inhibition area was observed around the bacterial zone of growth; (D) high antifungal activity: the *Bd* growth was only observed on the limits of the Petri plates; and (E) strong antifungal activity: no *Bd* growth was detected. A correlation analysis of anti-*Bd* activity of LABs was performed by using JMP Pro 12.1 software version (SAS Institute Inc.).

### Hydrophobicity of the bacterial surface

We determined the hydrophobicity and Lewis acid/base properties of LABs by the Microbial Adhesion to Hydrocarbon (MATH) assay [73] using different organic solvents: xylene (apolar), chloroform (electron acceptor) and ethyl acetate (electron donor). The LABs were grown in MRS broth as indicated above, collected by centrifugation (3,000 *g*, 4°C) at the early logarithmic growth phase (7 h), washed twice and resuspended by using sterile distilled water to an Optical Density (OD_600 nm_) of 0.6. Chloroform, ethyl acetate and xylene (0.45 mL) were added to test tubes containing washed cells (2.7 mL). The samples were gently shaken in a vortex for 90 s. The tubes were left to stand for 15 min for separation of the both organic and aqueous phases. Then, the aqueous phase was separated with a 1000 μL micropipette and the OD was determined using a Shimadzu spectrophotometer (Shimadzu Corporation, Japan). The hydrophobicity was calculated using the following formula: % Hydrophobicity = [(OD before mixing-OD after mixing)/OD before mixing] x 100. The degree of bacterial hydrophobicity was classified as low (0–29%), medium (30–59%) or high (60–100%).

### Exopolysaccharide (EPS) production and biofilm formation

We studied the EPS synthesis by LABs using the Congo red agar method [74]. The culture medium contained (in g/L): brain heart infusion, 37; sucrose, 50; agar, 10; and Congo red, 0.8. The stain was prepared as a concentrated aqueous solution and autoclaved separately (121°C, 15 min), while sucrose was sterilized using 0.2 μm Millipore membranes. Both stain and sucrose were added when the agar medium achieved 45°C. Plates were inoculated with 10 μL of 1x10^5^ CFU/mL of each LABs and incubated for 48 h at 37°C in microaerophilic conditions. The presence of a dark blue microbial growth indicated that the isolate was an EPS producer. *Lactobacillus casei* CRL 87 was used as positive control [75].

The biofilm formation was assayed in each LABs using the crystal violet-stained microplate assay [76]. In brief, bacterial cells from the third subculture in MRS medium without Tween 80 were washed and resuspended in PBS solution pH 6.8 to get an OD_540 nm_ of 1.2 (~ 4x10^8^ CFU/mL). We then took a 100 μL bacterial suspensions and inoculated it into 2.5 mL MRS broth without Tween 80, an inhibitory surfactant of biofilm formation [76], and 200 μL aliquots were added to 96-well polystyrene microplates that were incubated under static conditions at 37°C for 72 h. Wells were washed three times using PBS solution at pH 6.8 and the quantification of the biofilm formed was carried out according to Leccese Terraf et al. [59,76]. Briefly, 200 μL crystal violet (0.1%) were added to the wells that were washed as indicated above after 15 min of co-incubation. The biofilm was detached using 200 μL absolute ethanol and quantified by measuring the OD_540 nm_. Additionally, sterile culture medium was included as negative control. For biofilm quantification, a cut-off (ODc) was defined as the mean OD value of the negative control. Based on the OD values obtained, LABs were classified as: negative (OD≤ODc), weak (ODc<OD≤ 2 x ODc), moderate (2 x ODc<OD≤ 4 x ODc) or strong (4 x ODc<OD) biofilm producers [77].

To interpret adhesion and colonisation properties of LABs, we carried out a multivariate correspondence analysis to evaluate the association between biofilm formation with EPS synthesis and auto-aggregation ability by using the InfoStat (2015p version) statistical software. For auto-aggregation, we used the data previously obtained in our research group [43]. All the assays mentioned above were performed in three independent trials and the average of the data were calculated and represented.

### Principal Component Analysis (PCA) of probiotic beneficial/probiotic properties of confirmed and presumptive lactic acid bacteria (LABs)

We carried out a PCA using JMP Pro software version 12.1 (SAS Institute Inc.) to determine and visualize the beneficial/probiotic properties of studied LABs including auto-aggregation ability [43], hydrophobicity, EPS synthesis and biofilm formation.

### DNA extraction and Random Amplified Polymorphic DNA (RAPD)-PCR analysis

For DNA extraction, LABs were grown in MRS broth as indicated above. Cells were recovered during the exponential growth phase (9 h) by centrifugation (3.000 x *g*, for 5 min at 4°C). Pellets were washed twice with sterile distilled water (SDW), fractioned in Eppendorf tubes containing 15 μL (SDW) and stored at -20°C. Then, cells were thawed, resuspended in 50 μL MilliQ water and microwaved at 700 W for 5 min [78].

The RAPD-PCR analysis is a simple and reliable method to assess DNA polymorphism. The ability to detect highly variable regions of DNA has application at the first stages of the bacterial species identification [79]. In this work, the M13 primer (5´GAGGGT GGCGGTTCT) [80] was used and the PCRs were performed in a TECHNE TC-512 thermocycler (Bibby Scientific, UK) under the following conditions: 5 min at 94°C of initial denaturation, 40 cycles consisting of 1 min at 94°C, 20 s at 45°C and 2 min at 72°C and a final extension at 72°C for 10 min. The RAPD reactions were carried out in a volume of 12.5 μL containing 3 mM MgCl_2_, buffer reaction (1x), dNTPs (200 μM each), 1 μM M13 primer, DNA (10–15 ng), and *Taq* DNA polymerase (0.1 IU; INBIO-Highway, Argentina). The RAPD products underwent electrophoresis at 100 V on a 2.5% agarose gel, stained with Gel Stain (Trans®, Beijing, China) and photographed under UV illumination. The RAPD-PCR patterns were grouped by means of cluster analysis with the Pearson product moment correlation coefficient and the unweighted pair group method using arithmetic averages (UPGMA). The RAPD band patterns (DNA fingerprint gel images) obtained were analysed using GelJ v.2.0 software [81] to obtain a dendrogram for both cocci and rods. Patterns with similarity values over 98% were considered genetically similar.

### Genotypic identification of selected beneficial microorganisms

From the RAPD-PCR analysis, we selected 16 pLAB for 16S rDNA sequence analysis by applying two criteria: 1) isolates with antifungal activity against at least two *Bd* isolates of different lineages and the Expression of one Adhesion/Colonisation-EAC property (hydrophobicity, auto-aggregation, EPS or biofilm production); 2) isolates without anti-*Bd* activity and the expression of at least two EAC properties. To identify and classify the selected pLAB as LAB, we amplified and sequenced the variable regions of the 16S rDNA gene. The isolates were grown as indicated above and DNA was extracted according to Pospiech and Neumann [82]. The reaction was performed with PCR buffer (1x) (Invitrogen, California, USA), 2.5 mM MgCl_2_ (Invitrogen, California, USA), 0.2 mM dNTPs (Invitrogen, California, USA), 1 μM MLB16 (5´GGCTGCTGGCACGTAGTTAG) and PLB16 (5´AGAGTTTGATCCTGGCTCAG) primers (used to amplify an ~500 bp region of the 16S rDNA gene, which contained the V1 and V2 variable regions) [83], *Taq* DNA polymerase (2.5 U) (Invitrogen, California, USA) and MilliQ water, to get a final volume of 50 μL. DNA amplifications were performed in a Bio-Rad MyCycler^™^ under the following conditions: 4 min at 94°C of initial denaturation, 30 cycles consisting of 30 s at 94°C, 45 s at 52°C and 45 s at 72°C and a final extension at 72°C for 7 min. The PCR products were electrophoresed in 1% agarose gels, purified and sequenced using the DNA sequencing service of CERELA (Tucumán, Argentina). Identification was performed by comparing the obtained 16S rDNA sequences with those deposited in Genbank database using the BLAST algorithm and considering a percentage of identity ≥98% (e-value over 98% is = 0 in a fragment of 500 bp) as traditional species level cut-off.

### Compatibility assays

To determine if the selected microorganisms could be included in a mixed probiotic product, we carried out compatibility assays among 18 LAB: *E*. *gallinarum* CRL 1826, *L*. *garvieae* CRL 1828 and the 16 LAB identified from 16S rDNA sequences analysis using the agar-well diffusion method [40,41,43]. All LAB were grown in TG broth for 9 h and the crude supernatants were used to determine its inhibitory effect. Compatibility assays consisted of 1x10^6^ CFU/mL of one LAB (potentially indicator isolate) in five Petri plates containing soft TGA (0.7% w/v) that were punched to create 6 holes (10 mm each). Then, 100 μL of crude supernatants from each of the other 17 LAB (antagonistic metabolite producer isolates) were added to each well. Moreover, TG broth was used as a negative control. The presence of an inhibitory halo of the bacterial growth indicated that the isolates were not suitable for combining in a mixed probiotic consortia.

## Results

### Anti-*Batrachochytrium dendrobatidis* (*Bd*) activity of confirmed and presumptive lactic acid bacteria (LABs)

**Fig 1:**
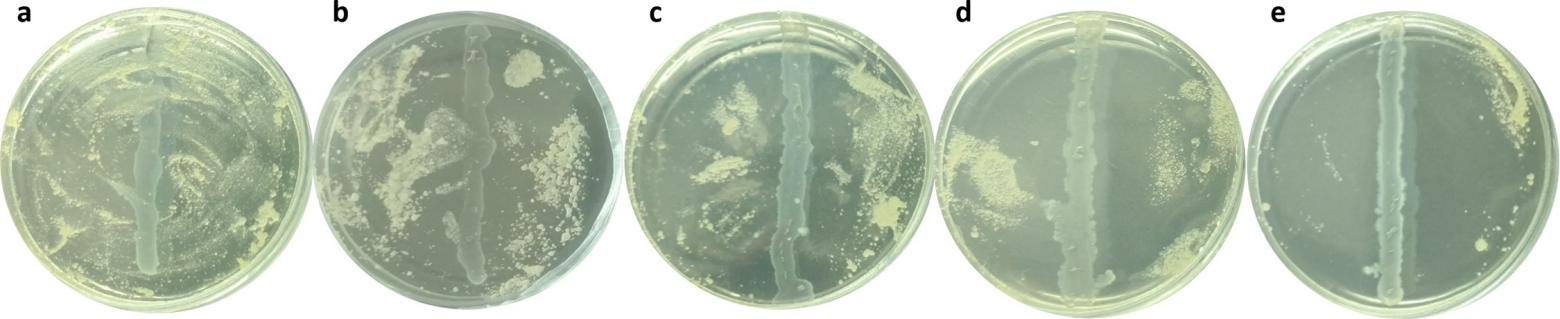
Score of *in vitro* anti-*Batrachochytrium dendrobatidis* (*Bd*) activity of confirmed and presumptive lactic acid bacteria isolated from North American bullfrog (*Lithobates catesbeianus*). Anti-*Bd* scored as: **A)** Negative (pLAB: 3 vs. *Bd*: UM142), **B)** low (pLAB: 35B vs. *Bd*: CLFT001), **C)** medium (pLAB: 16B vs. *Bd*: VA02), **D)** high (*Enterococcus gallinarum* CRL 1826 vs. *Bd*: AVS7), and **E)** strong (pLAB: 45B vs. *Bd*: AVS7). pLAB: presumptive lactic acid bacteria.

The anti-*Bd* activity of studied LABs against eight *Bd* isolates is presented in **Table 3**. Most LABs did not show any anti-*Bd* activity; however several LABs inhibited all *Bd* isolates with a score between low to medium. Likewise, three *Bd*GPL and two hybrid isolates were highly inhibited by 10 LABs (**Fig 1D and Table 3**), while only two *Bd*GPL isolates were strongly inhibited by five LABs (**Fig 1E and Table 3**). On this basis, we established an inhibitory score of sensitivity from highest to lowest inhibition for the studied *Bd* isolates: AVS7>CLFT159>CLFT024.02>CLFT001>AVS4>VA02>UM142>C2A.

**Table 3:** Percentage and number of confirmed and presumptive lactic acid bacteria (LABs) with anti-*Batrachochytrium dendrobatidis* (*Bd*) activity on eight fungal isolates.

Anti-*Bd* score	Inhibition of *Bd* isolates (%)							
	*Bd*ASIA-2/*Bd*Brazil	*Bd*ASIA-2/*Bd*Brazil	*Bd*Hybrid	*Bd*GPL	*Bd*GPL	*Bd*GPL	*Bd*GPL	*Bd*GPL
	UM142	CLFT001	CLFT024.02	CLFT159	AVS4	AVS7	C2A	VA02
**Negative**	88.7;(55)	48.4;(30)	51.6;(32)	42;(26)	64.5;(40)	64.5;(40)	93.5;(58)	88.7;(55)
**Low**	6.4;(4)	25.8;(16)	25.8;(16)	19.3;(12)	30.6;(19)	12.9;(8)	3.23;(2)	6.4;(4)
**Medium**	4.8;(3)	22.6;(14)	12.9;(8)	25.8;(16)	3.2;(2)	12.9;(8)	3.23;(2)	4.8;(3)
**High**	-	3.2;(2)	9.7;(6)	9.7;(6)	1.6;(1)	4.8;(3)	-	-
**Strong**	-	-	-	3.2;(2)	-	4.8;(3)	-	-

The numbers of LABs that inhibited a specific *Bd* isolate is indicated between brackets.

**Fig 2:**
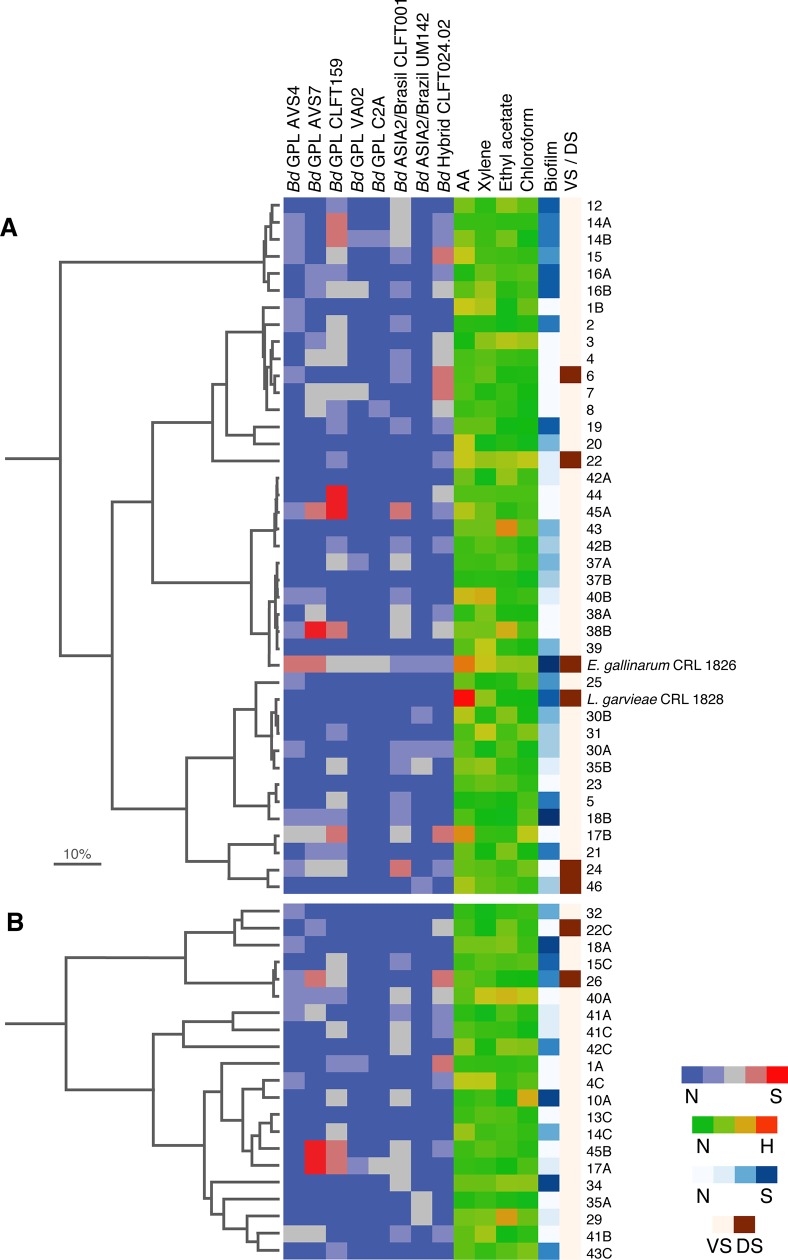
Summary of the results of probiotic/beneficial properties of confirmed and presumptive lactic acid bacteria (LABs). Dendrogram built based on PCR-fingerprint profiles: **A)** cocci, **B)** rods. We show information regarding the anti-*Batrachochytrium dendrobatidis* (*Bd*) activity, auto-aggregation, hydrophobicity and biofilm formation for LABs isolated from ventral (VS) and dorsal (DS) skin of bullfrogs. N, negative; S, strong; H, high.

We observed that approximately 67.8% of bacterial isolates did not inhibit *Bd* growth (e.g., pLAB: 3 against *Bd*: UM142), 16.3% showed low inhibition (e.g., pLAB: 35B against *Bd*: CLFT 001), 11.3% medium inhibition (e.g., pLAB: 16B against *Bd*: VA02), 3.6% high inhibition (e.g., *Enterococcus gallinarum* CRL 1826 against *Bd*: AVS7) and 1% strong anti-*Bd* activity (e.g., pLAB: 45B against *Bd*: AVS7) (**Fig 1**). A summary of the antimicrobial activity of LABs against *Bd* isolates of different lineages is shown in **Fig 2**.

*Enterococcus gallinarum* CRL 1826 was the most promising potential probiotic LAB since it inhibited the growth of all *Bd* isolates, with an efficacy ranging from low to high (**Fig 2A**). On the basis of the anti-*Bd* activity we also selected some tested pLAB as probiotic candidates. They include cocci 38B (medium inhibition against two hybrids, and from high to strong inhibition for two *Bd*GPL isolates), 17B (medium to high inhibition against both two hybrids and three *Bd*GPL isolates), 35B (medium inhibition against one hybrid and one *Bd*GPL isolate) and 45A (from high to strong inhibition against one hybrid, and two *Bd*GPL isolates, respectively) (**Fig 2A**). Among the rods, we selected the pLAB 1A (high inhibition against one hybrid), 17A and 45B (medium inhibition against one hybrid and from high to strong inhibition against two *Bd*GPL isolates) (**Fig 2B**). Likewise, a partial correlation analysis used to evaluate the response of the *Bd* isolates when challenged with LABs revealed the pairs of *Bd* isolates that showed the highest similarity (i.e. higher positive associations) were VA02/C2A (0.5003), CLFT001/CLFT159 (0.4596) and AVS7/C2A (0.3604) (**Table 4**).

**Table 4:** Correlation analysis of anti-*Batrachochytrium dendrobatidis (Bd)* activity of confirmed and presumptive lactic acid bacteria (LABs).

*Bd* isolate	CLFT001	AVS4	AVS7	CLFT 159	VA02	C2A	UM142
**AVS4**	0.2359	.	.	.	.	.	.
**AVS7**	0.2174	0.1037	.	.	.	.	.
**CLFT 159**	**0.4596**	-0.1861	0.1442	.	.	.	.
**VA02**	-0.0331	-0.0622	-0.0927	0.0999	.	.	.
**C2A**	-0.1035	0.2405	**0.3604**	0.1301	**0.5003**	.	.
**UM142**	-0.0992	0.0499	-0.0484	-0.0364	0.0420	0.0787	.
**CLFT024.02**	-0.2273	0.2584	0.3185	0.2936	0.2819	-0.3526	-0.1466

### Hydrophobicity of the bacterial surface

Most of the LABs (90.3%) presented hydrophilic properties. For the hydrophobic isolates (9.7%), the mean values of adhesion to xylene, ethyl acetate and chloroform were 16.5%, 14.7% and 12.6% respectively. Some bacterial isolates showed both basic and acidic behaviour, such as *E*. *gallinarum* CRL 1826 and the pLAB 1B, 4C, 31, 39, 40A, and 40B, which exhibited a medium degree of hydrophobicity with xylene (nonpolar and acidic solvent; basic behaviour of the bacterial surface) as well as the pLAB 3, 10A, 10B, 17B, 22, and 40A with chloroform (monopolar and acidic solvent, basic bacterial surface properties). Likewise, pLAB 3, 22, 29, 38B, 40A, 43 and 42C demonstrated medium hydrophobicity using ethyl acetate (monopolar solvent; acidic bacterial surface character). From this group, we highlight isolate 40A, that adhered to all the solvents at the same level (37–43%) and this behaviour was likely due to the hydrophobic characteristics of its cell surface as observed for isolates 3 and 22, that adhered to chloroform and ethyl acetate (from 30.1 to 36.3%) (**Fig 2**).

### Exopolysaccharide (EPS) production and biofilm formation

**Fig 3:**
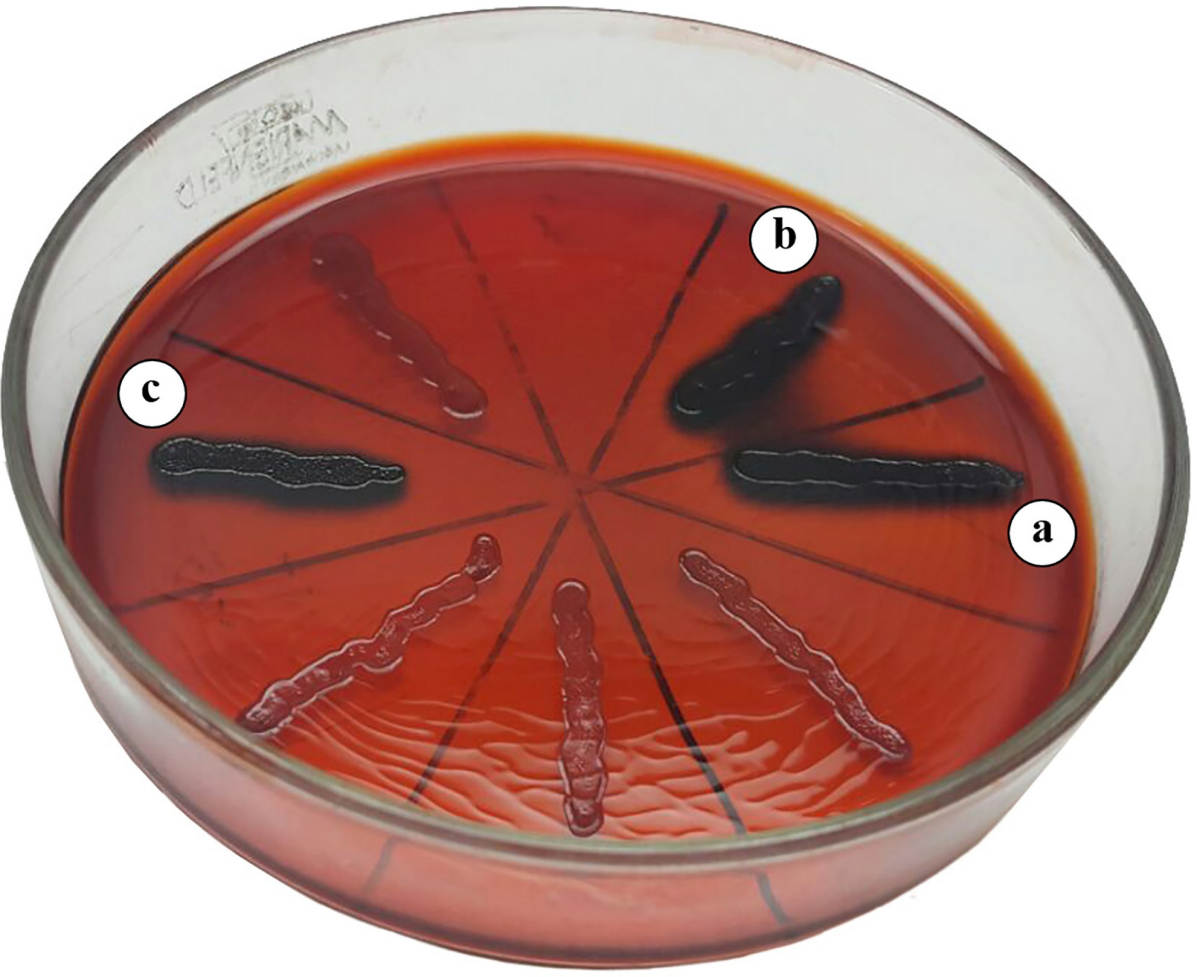
Exopolysaccharide production by confirmed and presumptive lactic acid bacteria. An EPS (+) isolate is indicated by the presence of a dark blue microbial growth line. **a)** *Enterococcus gallinarum* CRL 1826; **b**) pLAB 18B; **c**) *Lactobacillus casei* CRL 87 (positive control).

Our results show that 59.7% of LABs (n = 37) were EPS producers (e.g. *L*. *garvieae* CRL 1828, *E*. *gallinarum* CRL 1826 and the isolate 18B) (**Fig 3**). A total of 66.1% (n = 41) of LABs produced biofilm at different levels: 21% (n = 13) weak, 29% (n = 18) moderate, and 16.1% (n = 10) strong (**Fig 2**).

**Fig 4:**
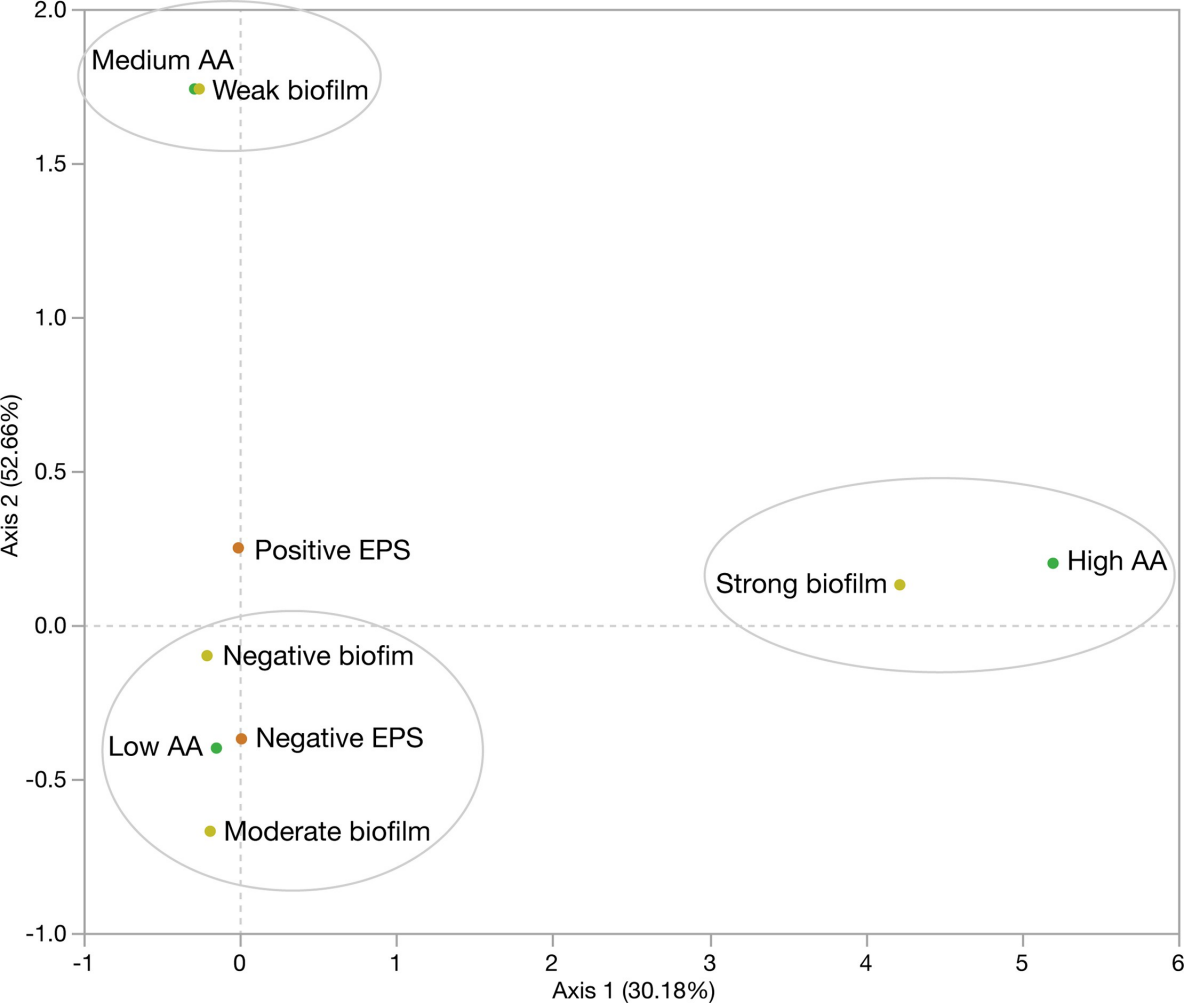
Analysis of correspondence of biofilm formation, exopolysaccharide synthesis and auto-aggregation by confirmed and presumptive lactic acid bacteria. The contribution to Chi-square is indicated in brackets.

Correspondence analysis investigating the interaction between biofilm formation, EPS and auto-aggregation, showed that negative and moderate biofilm producing LABs were associated with non-EPS producers and low auto-aggregating microorganisms. Likewise, strong biofilm formation was associated with high auto-aggregating LABs, while those with weak biofilm production were related to LABs with medium auto-aggregating capability (**Fig 4**).

### Principal Component Analysis (PCA) of probiotic beneficial/probiotic properties by confirmed and presumptive lactic acid bacteria (LABs)

**Fig 5:**
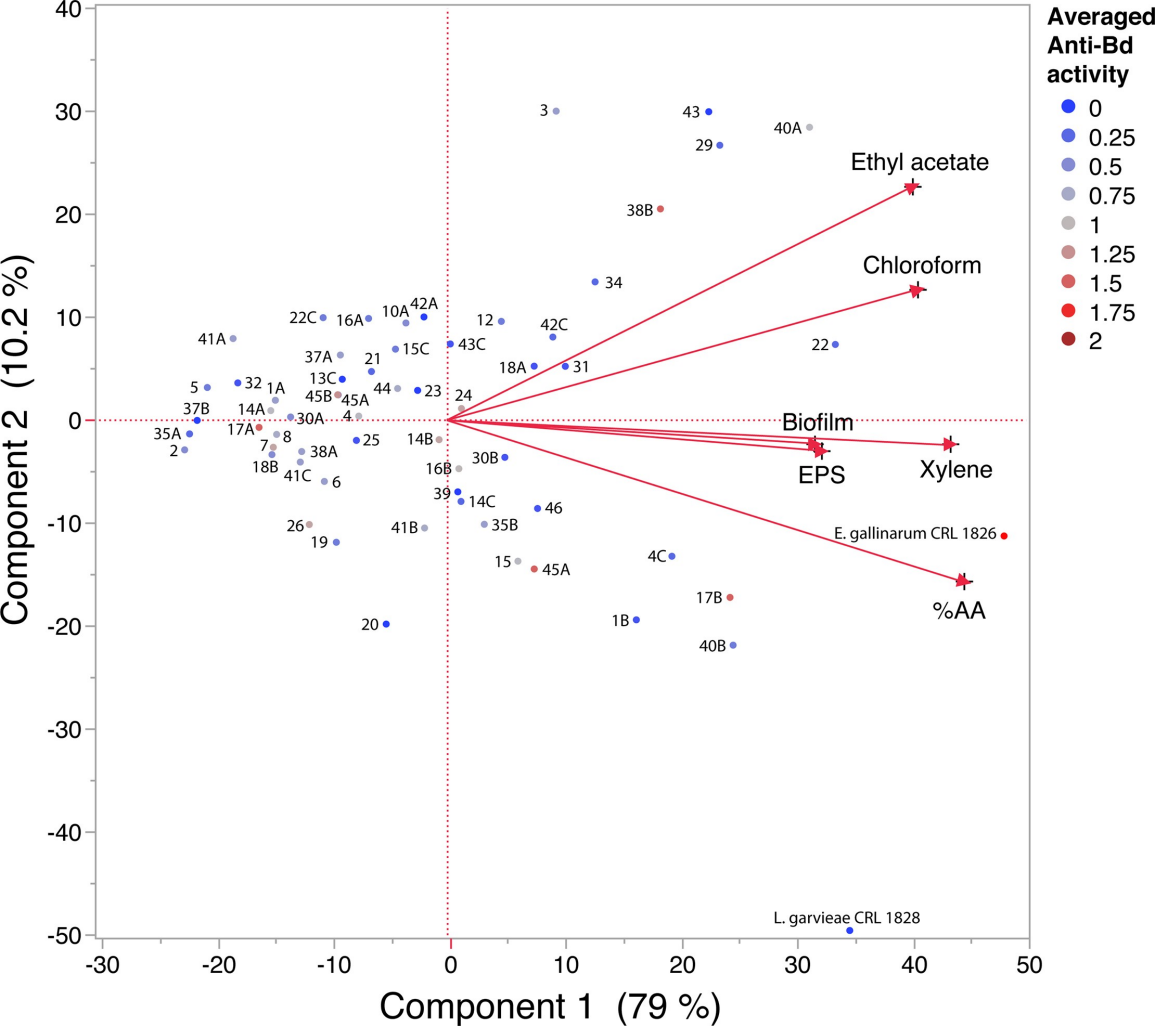
Principal Component Analysis (PCA) of beneficial properties (hydrophobicity in xylene, ethyl acetate and chloroform and AA-auto-aggregation, exopolysaccharide and biofilm formation) of confirmed and presumptive lactic acid bacteria (LABs). Averaged anti-*Bd* activity of every LABs for eight studied *Bd* isolates is shown by different colours (scored from 0: low inhibition for all studied *Bd* isolates to 4: high inhibition for all studied *Bd* isolates).

The first two components provided by the PCA explained 89.2% of the variation between the samples (79% Component 1 and 10.2% Component 2) (**Fig** 5). The biplot shows the results of the first two components (F1 and F2). Biofilm, EPS and hydrophobicity with xylene had high positive influence on component 1, while hydrophobicity with ethyl acetate and chloroform had a positive influence on component 2. Although the pLAB 40A and 22 (anti-*Bd* activity against *Bd*Hybrid and *Bd*GPL linages) displayed hydrophobicity, the last pLAB also showed auto-aggregation. Overall, *E*. *gallinarum* CRL 1826 exhibited the best profile of beneficial properties including the widest range of anti-*Bd* activity (**Fig 2**).

### Random Amplified Polymorphic DNA (RAPD)-PCR analysis and 16S rDNA gene sequencing

By considering a similarity pattern over 98% for LABs (n = 62), the genetic diversity among the cocci was 51% (21 different genotypes from 41 isolates) while for the rods was 90% (19 different genotypes from 21 isolates). Cluster analysis of RAPD-PCR patterns of LABs (41 cocci and 21 rods) revealed similarity values ranging from 54 to 100%. The cocci could be separated into two distinct main groups with similarity coefficient (SC) between 54–65%. The first main group (SC = 54%) included one subgroup with six genetically close isolates (SC = 97%), while the second main group (SC = 65%) included two subgroups, one comprising 22 isolates and a SC of 74%, and another group with 13 isolates and a SC of 81% (**Fig 2A**). The cluster analysis also demonstrated the presence of two principal groups of rod-shaped LABs with a SC of 55%. The first main group included two subgroups (SC = 81%) and three isolates each, while the second main group (SC = 74%) contained two subgroups with three and 12 isolates, respectively (**Fig 2B**). On the basis of RAPD-PCR results as well as established selection criteria, 16 pLAB (11 cocci and five rod-shaped) were selected for bacterial species identification by 16S rDNA gene sequencing analysis. The isolates were subsequently identified as *Pediococcus pentosaceus*, *Enterococcus thailandicus*, *Lactobacillus pentosus*/*L*. *plantarum*, *L*. *brevis*, and *L*. *curvatus* (**Table 5**). *Pediococcus pentosaceus* 15 and 16B were isolated from the same animal and showed similar beneficial properties. Likewise, *P*. *pentosaceus* 17B, 18B, 30A, 35B, 38B, 22 and 45A were isolated from different animals and expressed different probiotic characteristics. *Enterococcus thailandicus* 1B and 31, *L*. *pentosus*/*L*. *plantarum* 1A and 41A, *L*. *brevis* 40A and 41B were isolated from different animals and showed similar beneficial properties. Only one isolated was identified as *L*. *curvatus* 42C and showed surface properties and anti-*Bd* activity.

**Table 5:** Genetic identification of presumptive lactic acid bacteria (pLAB) using 16S rDNA sequence analysis.

pLAB	Animal	Identification	Beneficial properties	LAB identity (%)*	Accession number
1B	2	*Enterococcus thailandicus*	5; Hb; AA; (+)	*E*. *thailandicus* DSM 21767 **(99.78%)**	JXLE01000039
15	3	*Pediococcus pentosaceus*	2, 3, 4, 5; Hi, AA; (+); mB	*P*. *pentosaceus* DSM 20336 **(100.00%)**	JQBF01000022
16B	3	*Pediococcus pentosaceus*	2, 3, 4, 5, 8; Hb; (+); mB	*P*. *pentosaceus* DSM 20336 **(100.00%)**	JQBF01000022
17B	3	*Pediococcus pentosaceus*	2, 3, 4, 5, 6; Hb; AA; (+)	*P*. *pentosaceus* DSM 20336 **(99.57%)**	JQBF01000022
18B	6	*Pediococcus pentosaceus*	3, 4, 5, 6; Hi; (+); sB	*P*. *pentosaceus* DSM 20336 **(99.58%)**	JQBF01000022
22	12	*Pediococcus pentosaceus*	2, 3; Hb; AA	*P*. *pentosaceus* DSM 20336 **(99.37%)**	JQBF01000022
30A	7	*Pediococcus pentosaceus*	1, 2, 4, 5; Hi; wB	*P*. *pentosaceus* DSM 20336 **(99.57%)**	JQBF01000022
31	3	*Enterococcus thailandicus*	3; Hb; (+); wB	*E*. *thailandicus* DSM 21767 **(98.91%)**	JXLE01000039
35B	6	*Pediococcus pentosaceus*	1, 3, 4; Hb; (+)	*P*. *pentosaceus* DSM 20336 **(99.57%)**	JQBF01000022
38B	8	*Pediococcus pentosaceus*	2, 3, 4, 5, 6; Hb	*P*. *pentosaceus* DSM 20336 **(99.58%)**	JQBF01000022
45A	4	*Pediococcus pentosaceus*	2, 3, 4, 5, 6; Hi; AA	*P*. *pentosaceus* DSM 20336 **(99.78%)**	JQBF01000022
1A	2	*Lactobacillus pentosus/ Lactobacillus plantarum*	2, 3, 8; Hi; (+)	*L*. *pentosus* DSM 20314/ *L*. *plantarum* strain OZD95-42 **(99.78/99%)**	AZCU01000047
40A	2	*Lactobacillus brevis*	2, 3, 4, 5, 6; Hb	*L*. *brevis* ATCC 14869 **(99.36%)**	MK333777.1
41A	21	*Lactobacillus pentosus/ Lactobacillus plantarum*	2, 4, 5, 6; Hi; (+)	*L*. *pentosus* DSM 20314/ *L*. *plantarum* strain RKB18-46 **(99/99%)**	KI271266
41B	21	*Lactobacillus brevis*	2, 4, 5, 6; Hb	*L*. *brevis* ATCC 14869 **(99.14%)**	AZCU01000047
42C	3	*Lactobacillus curvatus*	4; Hb; AA	*L*. *curvatus* JCM 1096 **(100.00%)**	MK333781.1

Anti-*Bd* activity against: 1-UM142, 2-CLFT024.02, 3-CLFT 159, 4-CLFT001, 5-AVS4, 6-AVS7, 7-C2A and 8-VA02. Hi: hydrophilic; Hb: medium hydrophobicity; AA, auto-aggregating; EPS producer: (+); Biofilm formation: weak (wB), moderate (mB), and strong (sB).

*LAB identity (%): % of identity between the sequence under study and those incorporated in the database.

### Compatibility assays among selected lactic acid bacteria

Since LAB showed broad-spectrum inhibition across a range of *Bd* isolates, there is potential to design a multi-strain probiotic that may be effective in mitigating chytridiomycosis outbreaks. To this end, we performed compatibility assays among the 18 LAB that included *E*. *gallinarum* CRL 1826, *L*. *garvieae* CRL 1828 and the 16 identified LAB listed in **Table 5.** From our compatibility results, we did not observe any inhibitory halos (data not shown), indicating that all LAB can be combined in the design of a mixed probiotic formula.

## Discussion

Considering that Gram-negatives have been proposed as probiotics owing to their high *Bd* inhibitory activity against *Bd*GPL isolates [21,84,85], that LAB belong to the native microbiota of bullfrogs [39–41,43] and known safety of certain species [48], we evaluated the following physiological parameters of LAB to assess their suitability as probiotics: 1) the *in vitro* anti-*Bd* activity of LABs isolated from bullfrog skin against several *Bd* isolates from diverse genotypes, some of them related to amphibian declines [15], and 2) properties related to host cell adhesion and colonisation. We demonstrate that antifungal activity of tested LABs varied based on *Bd* isolate, even among closely related *Bd* genotypes such as AVS4 and AVS7 [71]. The C2A (*Bd*GPL) was the least sensitive isolate in terms of LAB inhibition. Likewise, *E*. *gallinarum* CRL 1826 demonstrated promise as a probiotic candidate for *Bd* control since it showed medium to high anti-*Bd* activity against all *Bd*GPL isolates. However, the CRL 1826 strain showed low inhibition of *Bd* hybrids. Conversely, pLAB 17B and 35B inhibited *Bd* hybrids to a medium degree. These findings support those of Antwis and Harrison [86] who showed that inhibition of different *Bd*GPL isolates by a single bacterial strain is unusual. The diverse *Bd* inhibitory patterns exhibited by LABs may be explained by the fact that both kind of microorganisms were isolated from different amphibian species and geographical regions. Muletz-Wolz et al. [27] found that in *Bd-*negative salamander species, a small number of anti-*Bd* bacterial strains were present on multiple host species at various localities, but none were shared among all species and localities, indicating the strong influence of the environment over the structure of bacterial skin communities. It was also found that bullfrogs can harbour one of the less sensitive *Bd*Hybrids: UM142 [66], but also LABs [39–41,43]. Therefore, we can hypothesize that some LAB and the UM142 isolate could have coevolved by developing resistance to some antimicrobial compounds produced by the prokaryote group.

The *in vitro* anti-*Bd* activity of LABs observed in this study could be attributed to antagonistic metabolites (hydrogen peroxide, organic acids, aroma compounds, and/or bacteriocins) that diffused from the bacterial growth streak. Hydrogen peroxide generates oxidative stress and affects cellular signalling pathways [87], while the organic (lactic and acetic) acids exert their antimicrobial action after penetrating cell membranes in their undissociated form, leading to a drop in the intercellular pH and to the disruption of metabolic activities [88]. It has been reported that hydrogen peroxide alone or combined with both, acetic and peracetic acids, inhibits *Bd* growth [89], while bacteriocins and volatile organic compounds may be responsible for *Bd* inhibition [90]. We hypothesize that some of the metabolic end-products (alone or combined) synthesized by LABs, and probably some of those cited in **Table 1**but not studied in LAB, would be responsible of the anti-*Bd* activity, and the mechanism of inhibition would depend on both LABs and each particular *Bd* isolate/lineage. However, competitive exclusion during the *in vivo* assays should not be discarded, especially when the potentially probiotic microorganisms have properties related to adhesion and colonisation processes.

The microbial surface plays an important role in how microbes interact with other microorganisms and the environment, mainly through adhesion to bacteria, eukaryotic cells [91] and other surfaces that allow colonisation of different ecosystems/hosts [92,93]. The mechanisms of bacterial adhesion include electrostatic and hydrophobic interactions (low affinity mechanisms) for which the acidic or basic characteristics (polarity) of the cell surface have a relevant role and can be used as criteria to predict adhesion capability [73,94]. Since hydrophobicity of the bacterial surface may be related to bacterial growth on hydrophobic substrates, auto-aggregation, biofilm formation and adhesion to host cells [57,95], this surface property must be considered as a relevant criterion for probiotics selection. The high proportion of hydrophilic microorganisms found in our experiments could be related to both the aqueous environment and the chemical nature of the mucus [96], and support the reported results for LAB isolated from another bullfrog hatchery in Argentina [39,41]. Hydrophilic LABs appear to be suitable probiotic candidates since they adhere better to the host mucus than epithelium, and would eliminate *Bd* by competitive exclusion and/or anti-*Bd* activity together with other components of the mucosome [14]. Considering that *Bd* is a keratinophilic pathogen [97] and thus must go through the mucus barrier to reach the outer epidermal layers, hydrophobic LABs may be relevant because they can adhere to the skin epithelial cells and inhibit fungal infections by specific blockage of cell receptors or inhibiting host attachment by steric interactions. Our hydrophobic LABs showed different patterns of surface polarity (acidic or basic). In *Lactobacillus* strains it has been shown that following an initial nonspecific contact with host epithelial cells, specific interactions occur between specialized molecules (adhesins) and epithelial cell receptors, in addition to S-coat proteins [98]. Therefore, based on our findings we propose that *E*. *gallinarum* CRL 1826 and pLAB 17B (medium hydrophobicity, auto-aggregating, anti-*Bd* activity), *L*. *garvieae* CRL 1828 (hydrophilic, auto-aggregating, without anti-*Bd* activity), the pLAB: 22, 29, 35B, 38B, 40A, and 40B (medium hydrophobicity, with anti-*Bd* activity), 43 (medium hydrophobicity, without anti-*Bd* activity), and 1A, 15, 18B, 30A, 41A and 45A (hydrophilic, with anti-*Bd* activity) may be potential probiotic candidates.

Bacterial surface polysaccharides are considered key macromolecules in determining microbe-host interactions through passive forces, electrostatic interactions, and hydrophobic and steric forces [99,100]. Surface polysaccharide production is widely reported in LAB isolates, in particular among members of the *Lactobacillus* genus [101,102]. Since polysaccharides display a high diversity among LAB [103], they are thought to be involved in determining relevant strain-specific properties for probiotic action, such as the degree of bacterial adhesion to host cells [104–106,60]. We evaluated EPS synthesis by LABs to inform the selection of potentially probiotic isolates with the goal of obtaining a high proportion of EPS producers. This beneficial property and those cited above, support the selection of *E*. *gallinarum* CRL 1826 and the pLAB 1A, 15, 18B, 40B, and 41A as potential probiotic candidates. Recently, Ringø et al. [52] established that live LAB from fish aquaculture can produce bioactive compounds such as EPS that maintain the natural state of microbe-associated molecular patterns (MAMP) structures contributing to the superiority of immunostimulant effects over the inactivated form of LAB.

Biofilm formation by LAB promotes mucosal colonisation and can mask epithelial cell receptors by preventing pathogen adhesion by competitive exclusion [107,108]. Our results indicate that approximately 45% of tested LABs were moderate/strong biofilm producers. This ability, together with the anti-*Bd* activity and surface properties, suggest the following bacterial isolates in the selection of probiotic candidates: *E*. *gallinarum* CRL 1826, *L*. *garvieae* CRL 1828 and the pLAB 15, 18B and 43. Although bacterial aggregation and EPS synthesis have a role on biofilm formation [109,110], our correspondence analysis indicated that biofilm synthesis was LABs specific with high aggregating bacteria correlating with strong biofilm producers, and EPS producers being correlated to negative biofilm producing LABs. With respect to aquaculture, Lamari et al. [111] reported biofilm production on abiotic surfaces in potentially probiotic *Lactobacillus casei* strains from *Artemia* sp. cultures and proposed the possible ability of LAB to colonise the gut, and to further antagonize pathogens.

Our RAPD-PCR results for LABs indicated a higher degree of genetic diversity among the rods than the cocci when using similarity patterns over 98%. However, other authors have been less rigorous and reported that similarity patterns over a reproducibility level of 83 and 85% were considered genetically similar for LAB from fermentation processes [112,113]. In line with our specified criteria, we selected 16 pLAB (cocci and rods) for 16S rDNA sequencing that allowed us to classify them as LAB, with *E*. *thailandicus* being reported for the first time from bullfrog skin, but not the other identified species [39,41].

Interestingly, the same genus and species identified in this work has been isolated from different bullfrog specimens and skin (ventral and dorsal) areas. These findings could be explained by the life-history of hosts, which carry out their biological cycle in aquatic and terrestrial environments, facilitating an ongoing microbiota exchange. Although we detected differences in the probiotic characteristics expressed by isolates of the same genus and species, these are likely due to strain-specific factors. Additional studies are therefore required to identify LAB species at the strain level.

The use of microbial consortia in probiotic formulations would provide advantages over single microorganisms, in part due to the wider range of beneficial functions conferred by a community [114–116]. Our results indicate that all LAB selected as probiotic candidates can be used in the design of a mixed formula, effective against all *Bd* isolates studied in this work. In this bacterial consortium, each bacterial strain would participate with a specific probiotic property (anti-*Bd* activity against one or more *Bd* lineage, and/or characteristics related to adhesion and colonisation) that could potentially act in synergy.

The experimental framework presented here represents the basis to select LAB as probiotics for *Bd* control, but other *in vitro* assays such as adhesion to keratin and resistance to amphibian antimicrobial peptides present in the skin mucus must be performed to select suitable LAB for *in vivo* studies. Considering that bacteria with anti-*Bd* activity have had variable success for *Bd* control by bioaugmentation [27], experimental assays with selected LAB strains (alone or in bacterial consortia) must include different amphibian species from diverse geographical regions to guarantee their effectiveness. These studies will allow us to determine long-term persistence of the administered LAB and to propose a specific protocol of LAB administration for a particular amphibian species.

## Conclusion

This is the first report on skin-associated LAB from bullfrogs to advance in the design of a probiotic product with applications in *Bd* control and mitigation. Our analysis of anti-*Bd* activity and adhesion/colonisation properties have allowed us to select 18 LAB, with *E*. *gallinarum* CRL 1826, *L*. *garvieae* CRL 1828, *P*. *pentosaceus* 15 and 18B being the best probiotic candidates.
